# The effects of visitors and social isolation from a peer on the behavior of a mixed-species pair of captive gibbons

**DOI:** 10.1038/s41598-022-23196-8

**Published:** 2022-11-16

**Authors:** Saein Lee, Heungjin Ryu, Yoonjung Yi, Seon-a Jang, Haeun Gye, Ahyun Choi, Haeun Cho, Bae-keun Lee, Jae C. Choe

**Affiliations:** 1 Laboratory of Behaviour and Ecology, Interdisciplinary Program of EcoCreative, Ewha Womans University, Seoul, 03760 Republic of Korea; 2 School of Life Sciences, Ulsan National Institute of Science and Technology, Ulsan, Republic of Korea; 3 Department of Basic Research, National Institute of Ecology, Seocheon-Gun, Republic of Korea; 4 Laboratory of Animal Behaviour and Conservation, College of Biology and the Environment, Nanjing Forestry University, Nanjing, 210037 Jiangsu China; 5 Division of EcoScience, Ewha Womans University, Seoul, 03760 Republic of Korea; 6 Department of Biological Sciences, Pusan National University, Busan, Republic of Korea; 7 Department of Zoological Management, National Institute of Ecology, Seocheon-Gun, Republic of Korea; 8 Administration Bureau, Division of Social Responsibility Support, National Institute of Ecology, Seocheon-Gun, Republic of Korea

**Keywords:** Behavioural ecology, Animal behaviour

## Abstract

Human visitors affect the behavior of captive animals, which is the so-called visitor effect. The number and behavior of visitors may influence stress-related behaviors in captive animals, such as self-scratching, yawning, and visitor-directed vigilance. A social group setting can be applied to alleviate such negative visitor effects and facilitate social behavior and interactions between individuals. In this study, we examined how the number and behavior of visitors are related to stress-related behaviors of a captive mixed-species gibbon pair comprising a yellow-cheek gibbon (*Nomascus gabriellae*) and a white-handed gibbon (*Hylobates lar*). The two gibbons were separated during the study period, and we examined whether the social isolation stimulated the visitor effect. The frequency of stress-related behaviors of the gibbons increased and the social playing between them decreased proportionally to visitor number. In the indoor enclosure, the gibbons increased their visitor-directed vigilance when visitors shouted or struck the glass partition. Our findings indicate that the number and behavior of visitors negatively affect captive gibbons and that a mixed-species social setting can help gibbons reduce visitor-induced stress. Future studies with larger sample sizes will improve the understanding of the visitor effect and the social setting in the captivity.

## Introduction

Public awareness of animal welfare in institutions such as zoos and care centers has grown^1^recently. Many researchers have studied the effects of a captive environment on animal behaviors^2,3^. Captive animals’ welfare is affected by not only individual-level factors, such as personality, genetics, and species characteristics, but also environment-level factors, such as the physical environment, social grouping, and enclosure type, which can influence the visitor effect^4–11^. The visitor effect, which is the influence of the presence and behavior of human visitors on captive animals, has been investigated for better management and species conservation. Although many studies have shown a negative visitor effect on captive animals^12–14^, some results show neutral or positive effects^5,15,16^. For example, captive chimpanzees (*Pan troglodytes*) solicited interaction with visitors^17^, their grooming increased, and stress-related behaviors decreased after positive interactions with keepers^18^.

An increase in the number of visitors (visitor density) is associated with a higher rate of visitor-directed vigilance and stress-related behaviors, including visitor- or conspecific-directed aggression, such as among cotton-top tamarins (*Saguinus oedipus*), Diana monkeys (*Cercopithecus diana diana*) and Western lowland gorillas (*Gorilla gorilla gorilla*)^5,19^. A previous study showed that higher visitor density also modifies the activity levels of captive primates. For example, chimpanzees (*Pan troglodytes*) spend less time on foraging, grooming, and playing^20^ and Diana monkeys (*Ceropithecus diana diana*) on grooming and resting^21^. Furthermore, a higher visitor density or increased visual contact with visitors may increase stress levels in captive animals. For example, the urinary cortisol levels of captive Colombian spider monkeys (*Ateles geoffroyii rufiventris*) elevated with increasing visitors^22^. In contrast, a decrease in the probability of visual contact between animals and visitors reduced glucocorticoid metabolite levels in black-capped capuchins (*Cebus apella*)^23^.

However, focusing solely on the visitor density cannot quantify the visitor effect^24,25^. Visitor behaviors, including talking, shouting, striking the glass partition, and feeding animals, are called visitor attention–getting behaviors (AGBs), which can attract captive animals^20^. One study conducted on 12 species showed that captive primates increased their locomotion responses toward more active visitors who tried to interact with them^26^.However, visitor density had no effect on their behavior. Previous studies have shown that the visitor behavior intensity (effect of visitor behavior on captive animals’ behavior) can have similar negative effects as the visitor density^27,28^. Although previous studies have coded the effect of visitor behavior intensity only dichotomously (e.g., passive [none of visitors attracted captive animals’ attention] vs. active [some visitors attracted captive animals’ attention])^5^, research is now considering various visitor behaviors in more detail. For example, the vigilance response of captive greater rhea (*Rhea americana*) increased with the increase of specific visitor behaviors, such as shouting, talking, and throwing food^29^. Aggressions from capped langurs (*Presbytis pileatus*), pigtailed macaques (*Macaca numestrina*) and olive baboons (*Papio anubis*) increased when visitors teased them and threw stones or sticks at them^30^. Considering these negative reactions of captive animals toward visitor behaviors, such as hitting and shouting, it is necessary to investigate the effect of specific visitor behaviors on captive animals in order to improve animal welfare^31^.

The social setting is one of the prominent variables that can alleviate negative visitor effects that affect animal welfare^32,33^. Captive animals can be housed in socially isolated conditions for easy management and/or avoiding aggression between individuals^34^. Consequently, they experience early social deprivation from their parents or peers, which sometimes results in depression, lack of social behaviors, self-directed behaviors, and neuroendocrinological issues^35,36^. Although previous studies have conducted social isolation for medical reasons, the results highlight the importance of the social setting by suggesting the negative effects of social isolation on captive animals’ behavior. Those negative effects may appear stronger in captive animals living in sympatry with conspecifics because animals’ social behavior can be shaped by interactions with conspecifics from early life^37^. As social isolation decreases opportunities for social interactions, it may limit the captive primates’ ability to handle visitor-induced stress through social interactions with conspecifics. Therefore, it is critical to consider the effect of the social setting when housing captive animals.

Primates who live together with conspecific individuals (social buffering) show stress alleviation^38,39^. Rhesus macaques (*Macaca mulatta*) exhibit less stress-related behaviors when socially housed compared to those housed alone^40,41^. Social buffering can also alleviate stress caused by previous social isolation^42^. The frequency of stress behaviors is negatively correlated with social behaviors when conspecifics or other captive members are present in the same enclosure, such as cotton-topped tamarins (*Saguinus oedipus oedipus*)^43^ and mangabeys (*Cercocebus galeritus chrysogaster*)^44^.

Gibbons have a pair-living social system and strengthen their social bonds through social grooming, playing and duetting^45,46^. Gibbons also have a high level of social tolerance toward conspecifics, which facilitates strong social bond formation between pair members^47^. Thus, social isolation might limit social interactions with conspecifics, which might affect gibbons’ capabilities of stress control^48^. Captive gibbons showed self-biting and visitor-avoidance behaviors as the number of visitors increased^49,50^. Therefore, we can expect a significant effect of social isolation, along with the visitor effect, on gibbons. Moreover, gibbons are sensitive to exposure to humans, showing increased self-directed behaviors and visitor-directed vigilance^51^. White-cheeked gibbons (*Nomascus leucogenys*) show self-directed behaviors more often with a large number of visitors, and male white-handed gibbons (*Hylobates lar*) show territorial behavior with teeth bearing more often with a large number of noisy visitors^52^. Family units of both siamangs (*Symphalangus syndactylus*) and white-cheeked gibbons (*Nomascus leucogenys*) spend more time in an area far away from the visitors’ viewing zone during days of large number of visitors^50^. In addition, early maternal separation and decrease in social contact with conspecifics increase sexual behaviors, such as masturbation, in eight *Hylobates* subspecies^53^. Given these negative reactions to visitors and social isolation of captive gibbons, providing practical solutions and guidelines by examining the visitor effect in relation to the gibbons’ social environment in captivity will contribute to their welfare.

In this study, we investigated the visitor effect in relation to a captive mixed-species gibbon pair comprising a yellow-cheek gibbon (*Nomascus gabriellae*) and a white-handed gibbon (*Hylobates lar*), which is unlikely to occur in nature. We investigated the effect of visitor behaviors, visitor density, and visitor behavior intensity; the effect of social isolation; and the combined effect of the visitor effect and social isolation on the captive gibbons’ behaviors. We hypothesized that increasing visitors’ density and behavior intensity negatively affect the behavior of captive gibbons. We predicted that (1) the visitor effect would affect social interactions by decreasing the spatial distance between the two gibbons to alleviate visitor-induced stress; (2) if the visitor density increases, the frequency of stress-related behaviors would increase and social playing would decrease; and (3) if visitors become more active (e.g., by shouting and striking the glass partition), the gibbons’ visitor-directed vigilance would increase. We also hypothesized that social isolation is negatively related to captive gibbons’ behaviors and will strengthen the negative visitor effect, as it also affects the social setting. We predicted that the two gibbons would exhibit increased stress-related behaviors and visitor-directed vigilance after social isolation. Our findings will contribute to suggesting pragmatic guidelines for managing visitors and social conditions that can reduce negative effects on captive gibbons.

## Methods

### Study subjects and site

From June to November 2018, we studied a pair of one female white-handed gibbon (*Hylobates lar*) and one male yellow-cheek gibbon (*Nomascus gabriellae*) housed at the Eco Care Center, National Institute of Ecology (NIE), Ministry of Environment, Seocheon, Republic of Korea, which specializes in caring for and protecting smuggled and illegally traded and endangered species found in Korea. The gibbons have been illegally traded when they were less than 2 years old. After being confiscated by the authorities, they were brought to the NIE in August 2016. Both gibbons exhibited sexual maturation in 2018, during the study period, so we estimated their age to be 6–8 years^54^.

### Housing conditions

**Figure 1:**
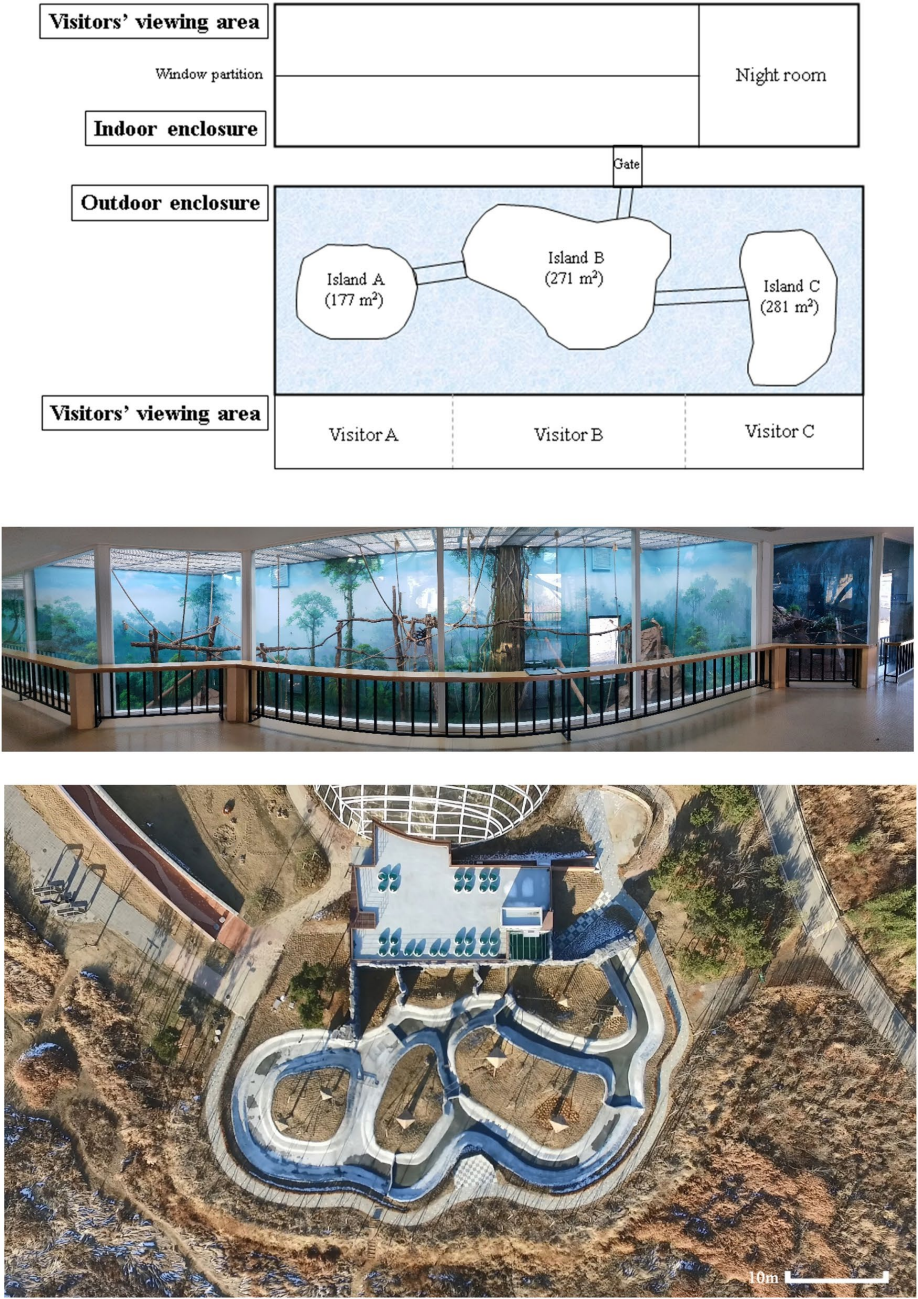
(Top) Aerial-view drawing of indoor and outdoor enclosures and the visitors’ viewing area. (Middle) Panoramic view of the indoor enclosure. (Bottom) Aerial view of the outdoor enclosure.

The outdoor enclosure at the NIE has three large main islands (island A: 177 m^2^; island B: 271 m^2^; island C: 281 m^2^), including an artificial shelter and trees, which are separated by ponds but connected via rope ladders for the gibbons to travel between islands (Fig. 1). The indoor enclosure (47 m^2^) has a glass partition that separates the enclosure from the public. The gibbons used both enclosures during the daytime (9:30 a.m. to 5:30 p.m.) and then were housed in a night room. The gibbons could move freely between the indoor and outdoor enclosures through a gate when they were housed together (further details of social isolation are described later). There is a window at the indoor enclosure, but two gibbons couldn’t see each other through this window due to the distance from the island of outdoor enclosure. All doors between the outdoor and indoor enclosure are closed after separation. Human visitors could observe the gibbons in both enclosures. The average temperature of the indoor enclosure was maintained at 22–26 °C, considering the natural habitat of gibbons (18–30 °C; NSW Agriculture, 2000). The gibbons were allowed to go to the outdoor enclosure when the lowest and highest temperatures were higher than 10 °C and 20 °C, respectively, but not when it rained hard. The caretakers fed each gibbon 800 g of fruits, vegetables, and primate food (Mazuri Leaf-Eater Primate Diet-Biscuit) in a day.

There are three phases of social isolation in captive gibbons. (1) non-isolation (from the beginning of June to the end of August 2018): gibbons housed together; (2) semi-isolation (from the beginning of September to September 27, 2018): gibbons partly separated; and (3) complete isolation (from September 28 to November 9, 2018): gibbons separately housed. When the caretakers observed gibbons exhibiting sexual behaviors, they were separated from each other (i.e., one individual in the indoor enclosure and the other in the outdoor enclosure, with the gate between the two enclosures being closed) to prevent potential interspecies contact during the daytime. Until the end of the study period, the gibbons were separated during the nighttime as well.

### Ethical notes

This study was conducted as part of the NIE management planning program and approved by the Institutional Animal Care and Use Committee (IACUC) of Ewha Womans University, South Korea. We used behavioral observation, which is a non-invasive approach. We maintained at least a 5 m distance from the gibbons in the outdoor enclosure and a 2 m distance in the indoor enclosure. Following the responsibility of NIE on prevention of interspecies hybrid, the two gibbon species were isolated from each other.

### Behavioral data collection

We collected data on human visitors and gibbons from 0930 to 1200 h and from 1400 to 1630 h for 27 days from June to November 2018. Specifically, we collected data on the visitor density (the number of visitors in front of indoor and outdoor enclosure of gibbons every minute), visitor behaviors (e.g., shouting at gibbons, striking the glass partition indoors), location of the gibbons (island ID), frequency of the gibbons’ self-directed behaviors (i.e., self-scratching, yawning), social-playing behavior (i.e., chasing the social-playing partner on the ground or while climbing the tree and rope), and visitor-directed vigilance (i.e., shaking the body or bipedal running with bared teeth toward visitors) using instantaneous focal sampling (1 min intervals for 20 min, n = 21 for each focal sampling session). We also collected data on the visitor density and the distance (in meters) between the two gibbons using scan sampling every 10 min (see Supplementary Table S1)^55^. This resulted in total of 270 scan samples of visitors.

We included both self-scratching and yawning as stress-related behaviors^56^ (Table 1). To determine the behavior direction, we analyzed visitor-directed vigilance toward visitors^57^. During the non-isolation and semi-isolation phases, we alternately observed each focal individual for focal sampling (20 min) and scan sampling (10 min intervals). For example, data was collected from the white-handed gibbon from 0930 to 1000 h and from the yellow-cheek gibbon from 1000 to 1030 h. During the complete isolation phase, we collected data from one focal individual per day: white-handed gibbon, 70 focal sampling sessions for 18 days; yellow-cheek gibbon, 50 focal sampling sessions for 21 days. During this phase, we did not record the frequency of social playing. To minimize bias between each other, a total of six observers discussed what the behavior is and decided on a consensus. Those observers collected data during the research period. Each day two observers recorded the same behavior data of each gibbon simultaneously.

**Table 1:** Ethogram of gibbon and visitor’s behaviors recorded using focal and scan sampling in the study.

Subject	Behavior	Description
Gibbon	Stress-related	Self-scratching: Using hand or foot for rubbing on the part of the body repeatedly. When gibbons stop rubbing, we considered it a single bout Yawning: Opening mouth widely; discriminated from normal yawning by showing more teeth with short intervals
	Visitor-directed vigilance	In both indoor and outdoor enclosure: Shaking or swaying body or bipedal running with bared teeth toward visitors. Making alarm call or noises toward visitors also counted in vigilance behavior In indoor enclosure: Rush to the window glass partition
	Social-playing	Playing with the other gibbon by chasing and mingling. When gibbons stop chasing, it is considered as a single play bout
	Shouting at gibbons	Shouting loud toward gibbons
Visitor	Striking the indoor enclosure	Knocking or hitting the window at indoor enclosure

*Each behavior is considered as a single bout if the behavior lasts for at least 5 s.

### Data analysis

#### Proximity and space use

We used R version 3.6.0 (R Development Core team) for statistical analysis. To determine whether visitor density affected the social interactions between the two gibbons, we used generalized linear mixed models (GLMMs) with negative binomial error distribution. First, we converted data from focal sampling session to behavior data per minute. Then, we *Z*-transformed the number of visitors to facilitate model convergence. We ran a negative binomial GLMM, with the distance between the two gibbons as the response variable, the number of visitors and the social isolation phase (only non-isolation and semi-isolation periods because we could not record the distance between the two gibbons during complete isolation) as explanatory variables, and the subject ID and date of data collection as random factors (model 1). To investigate the effect of visitor density on the two gibbons’ space use, we first calculated the proportion of each island being used (i.e., the percentage of a gibbon being on the island) during focal sampling (*n* = 3962 focal samples). Second, we compared this proportion when there was no visitor in front of each island (i.e., visitors A, B, and C in Fig. 1) to that when there was more than one visitor (mean ± SD = 10.53 ± 9.45) using chi-squared tests for each gibbon.

#### Stress-related behaviors and Social-playing

To estimate the effect of visitor density (the number of visitors) on the two gibbons’ stress-related behaviors, we again used GLMMs with negative binomial error distribution. First, we *Z*-transformed the number of visitors to facilitate model convergence. We ran a negative binomial GLMM, with the frequency of stress-related behaviors as the response variable, the visitor density and the social isolation phase as explanatory variables, the enclosure type (indoor/outdoor) as the control factor, and the subject ID and date of data collection as random factors (model 2a). We also included interaction between the visitor density and the social isolation phase and included the visitor density as a within-subject random slope. Second, to examine the effect of visitor density on the two gibbons’ play behavior, we ran a negative binomial GLMM, with the frequency of social playing as the response variable, the visitor density as the explanatory variable, the subject ID and date of data collection as random factors, and the visitor density as a within-subject random slope (model 2b). We excluded the social isolation phase in model 2b because we could not record social-playing behavior during the semi- and complete isolation phases. We also excluded the enclosure type as a control factor, because the gibbons were always together in the outdoor or the indoor enclosure during the non-isolation phase. When they were isolated, each gibbon used a different enclosure.

#### Effect of visitor behavior intensity

To determine the effect of the visitor behavior intensity (shouting at gibbons and striking the glass partition) on the two gibbons’ visitor-directed vigilance, we used GLMMs (models 3a and 3b) with negative binomial error distribution. In model 3a, we included the frequency of visitor-directed vigilance as the response variable, the frequency of visitors shouting and the social isolation phase as explanatory variables, and the subject ID and date of data collection as random factors. In model 3b, we ran another negative binomial GLMM, with the frequency of visitor-directed vigilance as the response variable, the frequency of striking the glass partition and the social isolation phase as explanatory variables, and the subject ID and date of data collection as random factors. We also included interaction between the frequency of visitor behavior and the number of visitors. We excluded the enclosure type as a control factor because the partition-striking behavior only occurred in the indoor enclosure.

We checked collinearity between explanatory variables using the *car* package^58^. The variance inflation factor (VIF) from all models was < 5. We used the *glmmTMB* package^59^ to run all GLMMs. We ran zero-inflation tests on the GLMMs using the *DHARMa* package^60^ and found that all models were not zero-inflated. Next, we ran null models, including only control factors, random effects (subject ID and date), and random slope (visitor density within the date). We obtained model estimates by using the *summary* function and confidence intervals (CIs) using the *confint* function. Finally, we conducted full-null model comparisons for each model using analysis of variance (ANOVA).

In all data analyses, we excluded the data point when the focal individual was not visible (18.2% of total data collection). The number of scan points of each model was as follows: model 2a (n = 3962), model 2b (n = 622), model 3a (n = 3962), and Model 3b (n = 2356). All full-null models were significantly better than null models (model1: *χ*^2^ = 10.369, *df* = 2, *p* < 0.01; model 2a: *χ*^2^ = 275.46, *df* = 4, *p* < 0.001; model 2b: *χ*^2^ = 15.63, *df* = 3, *p* < 0.001; model 3a: *χ*^2^ = 227.18, *df* = 2, *p* < 0.001; model 3b: *χ*^2^ = 332.19, *df* = 2, *p* < 0.001).

## Results

### Proximity and space use by gibbons

The distance between the two gibbons during the non-isolation phase was 2.29 ± 3.06 m (mean ± SD), while they were closer to each other during the semi-isolation phase (1.40 ± 2.39 m). There was no effect of visitor density, but social isolation affected the distance between the gibbons (Table 2). In addition, there was no relationship between visitor density and space use by the gibbons (white-handed gibbon: *χ*^2^ = 1.175, *df* = 2, *p* = 0.556; yellow-cheek gibbon: *χ*^2^ = 0.543, *df* = 2, *p* = 0.762). These results showed that the two gibbons used the islands in the outdoor enclosure in the same way regardless of the presence of visitors.

**Table 2:** Effects of visitor density and social isolation on the distance between gibbons (model 1).

	Estimate	SEM^a^	Lower CI^b^	Upper CI	*P*-value^c^
(Intercept)	1.057	0.480	0.257	1.857	**0.010**
Visitor density	0.116	0.107	− 0.093	0.325	0.277
Non-isolation vs. semi-isolation	− 1.411	0.727	− 2.836	0.015	0.052

*Visitor density (original mean ± SD = 3.38 ± 6.11); the reference level of the isolation phase is “non-isolation.”

^a^*SEM* standard error of the mean.

^b^*CI* confidence interval.

^c^*P*-values in bold are significant.

### Effect of visitor density and social isolation

**Figure 2:**
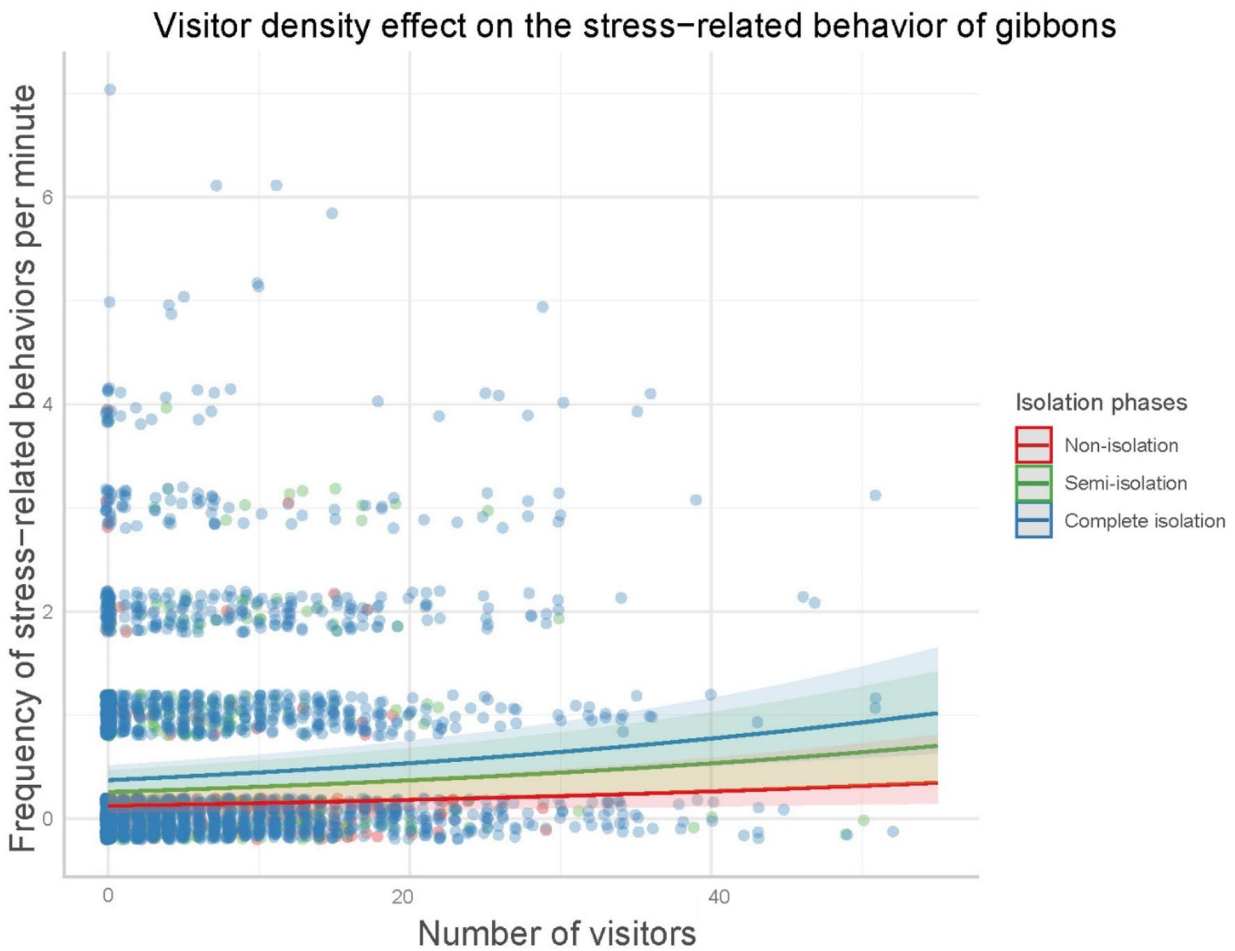
Effect of visitor density and social isolation (model 2a) on the stress-related behaviors of the two gibbons (*Hylobates lar* and *Nomascus gabriellae*). The shaded area indicates 95% confidence interval.

Visitor density and social isolation (model 2a) significantly affected the two gibbons’ stress-related behaviors (Table 3). Self-scratching and yawning were observed more frequently with increasing visitors during both semi-isolation and complete isolation phases compared to the non-isolation phase (Fig. 2). However, there was no effect of the enclosure type on stress-related behaviors.

**Table 3:** Effect of visitor density and social isolation on the stress-related behaviors of gibbons (model 2a).

	Estimate	SEM^a^	Lower CI^b^	Upper CI	*P*-value^c^
(Intercept)	− 1.970	0.379	− 2.713	− 1.227	** < 0.001**
Visitor density	0.142	0.029	0.085	0.199	** < 0.001**
Non-isolation vs. semi-isolation	0.703	0.381	− 0.043	1.450	0.065
Non-isolation vs. complete isolation	1.075	0.359	0.372	1.777	**0.003**
Visitor density × semi-isolation	0.429	0.210	0.018	0.840	**0.041**
Visitor density × complete isolation	0.299	0.194	− 0.080	0.935	**0.001**
Enclosure type	0.116	0.291	− 0.441	0.566	0.690

*Visitor density (original mean ± SD = 5.13 ± 7.74); the reference level of the isolation phase is “non-isolation.”

^a^*SEM* standard error of the mean.

^b^*CI* confidence interval.

^c^*P*-values in bold are significant.

**Figure 3:**
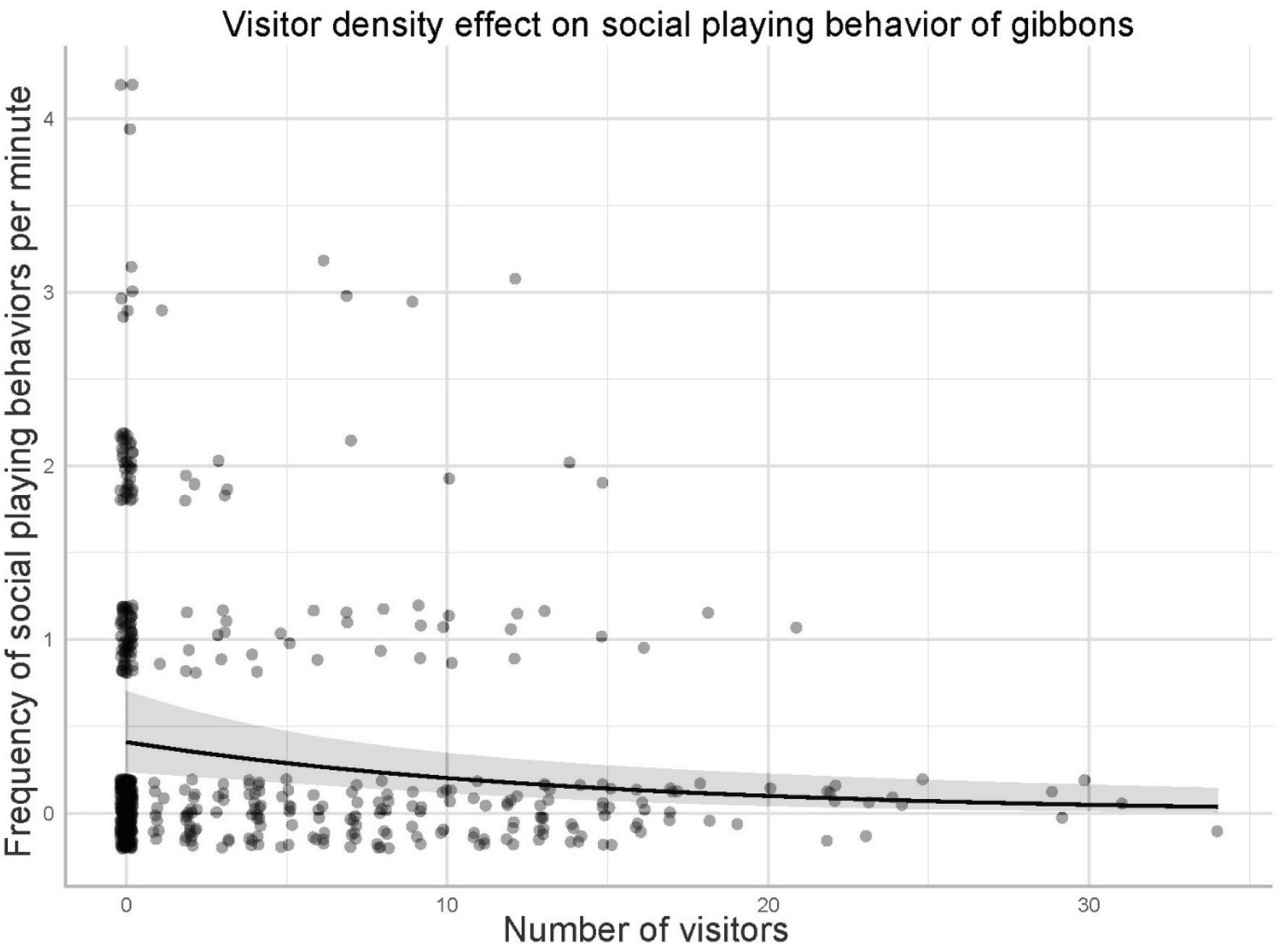
Effect of density effect (model 2b) on the social-playing behavior of the two gibbons (*Hylobates lar* and *Nomascus gabriellae*). The shaded area indicates 95% confidence interval.

In contrast, in model 2b, the frequency of social-playing behavior of the two gibbons decreased with increasing visitor density (Table 4 and Fig. 3).

**Table 4:** Effect of visitor density on social-playing behavior of gibbons (model 2b).

	Estimate	SEM^a^	Lower CI^b^	Upper CI	*P*-value^c^
(Intercept)	− 1.126	0.251	− 1.391	− 0.380	** < 0.001**
Visitor density	− 0.413	0.131	0.048	0.844	** < 0.001**

*Visitor density (original mean ± SD = 3.37 ± 5.91).

^a^*SEM* standard error of the mean.

^b^*CI* confidence interval.

^c^*P*-values in bold are significant.

### Effect of visitor behavior intensity and social isolation on visitor-directed vigilance

The frequency of visitor-directed vigilance increased when visitors shouted more often, with a marginally significant interaction effect between the frequency of visitor’s shouting and the number of visitors (model 3a; see Table 5). However, the effect of the visitor behavior intensity on visitor-directed vigilance did not change depending on social isolation and enclosure type (Table 5). In addition, the two gibbons displayed visitor-directed vigilance more often toward visitors who struck the glass partition more than who struck it less (model 3b; see Table 5).

**Table 5:** Effect of visitor behavior intensity (shouting) on the visitor-directed vigilance of gibbons (model 3a) and Effect of visitor behavior intensity (striking the glass partition) on the visitor-directed vigilance of gibbons (model 3b).

Model		Estimate	SEM^a^	Lower CI^b^	Upper CI	*P*-value^c^
Model 3a	(Intercept)	− 23.990	2609.807	− 5189.118	5091.138	0.993
	Frequency of visitors’ shouting (per min)	0.246	0.046	0.156	0.336	** < 0.001**
	Frequency of visitors’ shouting behavior (per min) × visitor density	− 0.007	0.004	− 0.014	0.001	0.069
	Non-isolation vs. semi-isolation	21.275	2609.807	− 5093.853	5136.404	0.994
	Non-isolation vs. complete isolation	20.195	2609.807	− 5094.932	5135.323	0.994
	Enclosure type	− 0.351	0.309	− 0.958	0.255	0.256
Model 3b	(Intercept)	− 24.953	2518.061	− 4960.262	4910.357	0.992
	Frequency of visitors’ partition-striking behavior (per min)	0.425	0.131	0.168	0.683	** < 0.001**
	Frequency of visitors’ partition-striking behavior (per min) × visitor density	− 0.010	0.010	− 0.030	0.010	0.3285
	Non-isolation vs. semi-isolation	22.680	2518.062	− 4912.630	4957.991	0.993
	Non-isolation vs. complete isolation	21.167	2518.061	− 4914.142	4956.476	0.993

*Visitor density (original mean ± SD = 5.53 ± 7.85); the reference level of the isolation phase is “non-isolation.”

^a^*SEM* standard error of the mean.

^b^*CI* confidence interval.

^c^*P*-values in bold are significant.

## Discussion

The visitor density negatively affects the behavior of captive gibbons, showing an increase in stress-related behaviors and decrease in social interactions proportional to the visitor number. Our result is consistent with previous research on white-handed gibbons (*Nomascus leucogenys*) in that increasing visitor density intensifies repeated self-scratching^52^. Self-scratching is a well-known measurement of stress and anxiety in both humans and nonhuman primates^61^. Self-scratching or yawning are well-known measurements of stress and anxiety in both human hand nonhuman primates^62^. However, few studies have investigated yawning as one of the probable variables to assess the effect of visitor density^62,63^. Our study combined both self-scratching and yawning as stress-related behaviors and found a positive correlation between them and the visitor density.

We also showed that an increase in visitor density reduced social playing between the two gibbons. This result is consistent with previous research on chimpanzees (*Pan troglodytes*) that showed decreased social playing with increasing visitor density^20^. Contrary to stress-related behaviors, such as self-scratching and yawning, social playing can decrease visitor-induced stress^2^. Previous studies on common marmosets (*Callithrix jacchus*) and squirrel monkeys (*Saimiri sciureus*) have shown that social playing can regulate and reduce stress^64^. Our study indicates that when the number of visitors increase, stress-related behaviors also increase but social playing decreases. Despite the stress-regulating function of social playing, visitor-induced stress may negatively affect social playing. Since the increase in visitors may increase the chance of interrupting social playing, the decrease in social playing can be a measure of stress (stress indicator), not a stress reducer^65^. Therefore, both stress-related and social-playing behaviors provide a better measure of visitor-induced stress in captive animals.

The two captive gibbons showed more vigilance directed toward visitors who shouted more at them and struck the glass partition more in comparison to visitors who did less shouting and striking. This result may support the previous finding that the visitor behavior intensity can be one of the causes of negative behaviors of captive animals^66^. Visitors’ AGBs may be a significant stress inducer which intensifies negative effects on captive animals’ behaviors. For example, there was a positive correlation between visitor-directed aggressions and the frequency of visitor behaviors such as offering objects or attempting to touch captive animals, but a negative correlation with visitor density in captive siamangs (*Symphalangus syndactylus*)^67^. In line with these studies, we found that visitor AGBs, including shouting and striking the glass partition, caused a negative response in the two gibbons. Measurement of such visitor behaviors can be a good indicator of the effect of visitor behavior intensity^12^.

Importantly, our result suggests that social isolation stimulates stress-related behaviors in captive gibbons, as the negative visitor effect was stronger during semi-isolation, indicating that social isolation may reinforce the visitor effect. Since the two gibbons experienced unique fostering conditions with peer rearing, social isolation might have had a greater effect on them compared to animals in other studies. Peer-reared pairs of captive rhesus macaques (*Macaca mulatta*), who grew up with peer conspecifics, not their parents, showed strong interdependence as in other pairs growing up^68^. Primates in social isolation may show stereotypical behaviors, including self-aggression, self-biting, self-clapping, stereotypical pacing, regurgitation, and even coprophagy^69,70^. Rehabilitation and modification of the social group composition can improve such stereotypical behaviors^36^. Although social isolation amplified the negative visitor effect on the two gibbons in this study, it had no effect on visitor-directed vigilance, which might be attributed to personality. For example, individual behavioral difference toward high visitor density were reported in Diana monkeys (*Cercopithecus diana diana*): some responded aggressively toward visitors, while others had an affiliative response^71^. Personality has been considered a vital indicator of animal welfare since many studies have found individual differences in the visitor effect^72^. Since non-conspecific pairing is not common in captive gibbons, it might lead to species-specific differences in the visitor effect. Another possible limitation of our study might be the small sample size (n = 2) and the short observation period of social isolation. Rhesus macaques (*Macaca mulatta*) socially isolated for a longer period felt more fear compared to those isolated for a short period^73^. Since we excluded 20% of data when gibbons were invisible, it also can be possible limitation of our study by missing their behaviors.

For better captive management, first, the negative effect of visitor density can be mitigated by regulating the number of visitors by time of day. Since in this study, both gibbons displayed more self-scratching and yawning and reduced social playing with increasing visitor density, it is crucial to control the number of visitors near the enclosure of gibbons. Informing visitors of the negative impact of their behaviors might also help reduce the negative effect of visitor behaviors. Practically, captive-animal managers can use signage that prohibit intense behaviors toward captive animals^74^. Second, remodeling enclosures in more naturalistic way also can regulate visitors’ intense behaviors^75^. Visual and auditory barriers can help captive animals be free from visual contact with visitors’ and noise^76^. With more free access to another enclosure or extra space to hide, captive animals display less stress-related behavior and visitor-directed vigilance^14,77^. Considering the arboreal lifestyle of gibbons, placing visitors below the gibbons, or providing shelters at a high place will free the gibbons from visual contact with visitors’^24^. Third, species-specific social factors, such as group composition and structure in the wild, should be integrated into captive management activities. Social relationship is important to alleviate visitor-induced stress in captive primates. Therefore, if possible, captive management activities that influence the group composition, such as social separation of animals, should be carefully performed. If social isolation is unavoidable, periodic monitoring of stereotypical and stress-related behaviors is necessary.

To sum up, visitor density, visitor behavior intensity, and social isolation can negatively affect captive gibbons’ behavior. Further study can use these factors as stressors to investigate the visitor effect and provide suggestions for alleviating stress in captive animals. More studies applying those suggestions will contribute to appropriate captive management leading to better welfare for captive animals.

## Supplementary Information

Supplementary Table S1.

## Data Availability

Data in support of the findings of this study are available from the corresponding authors by reasonable request.
