# Supplementary material for: The effects of visitors and social isolation from a peer on the behavior of a mixed-species pair of captive gibbons
Source: Sci Rep. 2022 Nov 16;12:19706. doi: 10.1038/s41598-022-23196-8 (PMC9668933; doi:10.1038/s41598-022-23196-8)
Supplement: Supplementary file 1 — Supplementary Table S1. [file 41598_2022_23196_MOESM1_ESM.docx]

# Supplementary Tables

**Table S1.** List of data collected on visitor and gibbon behaviors in the indoor and outdoor enclosures.

|  |  | Data collected at both outdoor and indoor enclosures | Data collected only at the indoor enclosure |
| --- | --- | --- | --- |
| Subject | **Gibbon** | Frequency of stress-related behaviors (self-scratching and yawning) | Frequency of visitor-directed vigilance |
|  |  | Frequency of social-playing |  |
|  |  |  |  |
|  | **Visitor** | The number of visitors (density) | Frequency of striking the glass partition (intensity) |
|  |  | Frequency of shouting at gibbons (intensity) |  |
